# Slow Food: Sustained Impact of Harder Foods on the Reduction in Energy Intake over the Course of the Day

**DOI:** 10.1371/journal.pone.0093370

**Published:** 2014-04-02

**Authors:** Dieuwerke P. Bolhuis, Ciarán G. Forde, Yuejiao Cheng, Haohuan Xu, Nathalie Martin, Cees de Graaf

**Affiliations:** 1 Division of Human Nutrition, Wageningen University, Wageningen, The Netherlands; 2 Sensory and Consumer Sciences, Nestlé Research Centre, Vers-Chez-Les-Blanc, Switserland; Paris Institute of Technology for Life, Food and Environmental Sciences, France

## Abstract

**Background:**

Previous research has shown that oral processing characteristics like bite size and oral residence duration are related to the satiating efficiency of foods. Oral processing characteristics are influenced by food texture. Very little research has been done on the effect of food texture within solid foods on energy intake.

**Objectives:**

The first objective was to investigate the effect of hardness of food on energy intake at lunch, and to link this effect to differences in food oral processing characteristics. The second objective was to investigate whether the reduction in energy intake at lunch will be compensated for in the subsequent dinner.

**Design:**

Fifty subjects (11 male, BMI: 21±2 kg/m^2^, age: 24±2 y) participated in a cross-over study in which they consumed ad libitum from a lunch with soft foods or hard foods on two separate days. Oral processing characteristics at lunch were assessed by coding video records. Later on the same days, subjects consumed dinner ad libitum.

**Results:**

Hard foods led to a ∼13% lower energy intake at lunch compared to soft foods (P<0.001). Hard foods were consumed with smaller bites, longer oral duration per gram food, and more chewing per gram food compared to the soft foods (P<0.05). Energy intake at dinner did not differ after both lunches (P = 0.16).

**Conclusions:**

Hard foods led to reduced energy intake compared to soft foods, and this reduction in energy intake was sustained over the next meal. We argue that the differences in oral processing characteristics produced by the hardness of the foods explain the effect on intake. The sustained reduction in energy intake suggests that changes in food texture can be a helpful tool in reducing the overall daily energy intake.

## Introduction

The rise in obesity over the last decades is considered to be related to changes in the food environment [1], [2]. Much of the current food supply consists of highly processed foods that support fast intake of energy and minimal oral processing, like energy-yielding beverages and softer solid foods [3]–[5]. Foods that can be consumed quickly may facilitate over-consumption. Many studies have shown that higher eating rate (grams or kilojoules per unit of time) leads to higher energy intake [6]–[10], and is related to higher body weight status [11]–[15].

The most obvious distinction in eating rate is between liquid and solid food forms [6]–[8]. Many studies have shown weaker satiating capacities of liquid foods compared to (semi)-solid foods [6]–[8], [16]–[20]. This effect has been attributed to the minimal sensory exposure of liquid foods in the oral cavity due to the fast rate of consumption [20], [21]. Very little research has been done on the impact of solid food textures on eating rate and energy intake. Investigating the effect of food texture on energy intake is relevant from a nutritional point of view, because (semi-) solid foods account for the majority (∼80%) of our daily energy intake [19].

The texture of a food is an important determinant of the eating rate [6], [9], [22]. Eating rate is negatively influenced by the duration of food spend in the oral cavity (oral residence duration) and positively by the bite size [9], [22]–[25]. Oral residence duration and bite size were shown to directly affect food intake in studies that used controlled experimental designs that keep eating rates (g/min) constant [23], [24], [26]. Increased chewing activity was also found to lower food intake [27], [28]. In these studies, oral processing characteristics have been altered explicitly [23], [24], [26]–[29], by giving instructions about chewing to subjects [27], [28], or by changing bite size and oral residence duration experimentally [23], [24], [26], [29]. The study of Forde et al. [22] compared oral-processing characteristics of a wide range of solid foods that were eaten in a natural manner. The results confirmed that foods that were consumed with smaller bites, higher chewing activity and longer oral residence duration per bite were expected to impart higher satiation [22].

In the present study, the textures of the foods were manipulated by using a “hard” and “soft” version of similar foods. The first objective was to investigate the effect of hardness on energy intake at lunch, and to link this effect to differences in food oral processing characteristics. The second objective was to investigate whether the reduction in energy intake will be compensated for at a subsequent meal at dinnertime.

## Subjects and Methods

### Subjects

Fifty-three subjects with Chinese nationality were recruited for participation, 50 (11 male) of whom completed the study. Two subjects dropped out before the start of the study, and one subject was excluded because instructions were not followed. Subjects were healthy as judged by themselves, they had a BMI of 21±2 kg/m^2^ (mean ± SD), and were aged between 20 and 29 y (24±2 y (mean ± SD)). Exclusion criteria were: following a vegetarian diet; following an energy-restricted diet during the last two months; gained or lost >5 kg weight during the last year; lack of appetite; food allergies or intolerances; difficulties with eating or swallowing. Subjects were informed that the research aimed to investigate the effect of food texture on the taste perception and palatability. Subjects were informed about the procedure of the study and signed an informed consent before participation. The study proposal was presented to the Medical Ethical Committee of Wageningen University, which decided that no formal approval was required. Subjects received a reimbursement after completion of the study. This study was registered (NTR: 3653) with the Dutch trial registration at: www.trialregister.nl/trialreg/admin/rctview.asp?TC=3653.

### Experimental design

This study was designed as a cross-over study in which subjects consumed ad libitum from a lunch with soft foods and a lunch with hard foods on two separate days in randomised order. Oral processing characteristics during lunch of the soft and hard foods were analysed by coding video recordings of subjects consuming the food. On the same day subjects came to consume dinner to investigate if differences in energy intake were compensated.

### Test foods

The lunch consisted of four hamburgers, which were composed of bread, meat, tomatoes and ketchup, and 600 g of rice salad (Table 1). The ingredients of one hamburger were: 45 g bread ( =  one bun, local bakery), 85 g hamburger meat (Mora, Tilburg, the Netherlands), ∼25 g tomatoes (2 slices), 20 g tomato ketchup (Heinz, Zeist, the Netherlands). The soft-hard manipulation was established by changing the type of bread (Table 1). The ingredients of the rice salad were: 300 g rice and 300 g vegetables (in pieces of 3–5 mm). The softer rice salad consisted of risotto rice (Lassie, Wormerveer, the Netherlands) and boiled vegetables and the harder rice salad consisted of white rice (Uncle Ben's, Zaventem, Belgium), and raw vegetables (Table 1).

**Table 1:** Lunch-items used for softer- and hard foods.

Test foods	Ingredients	Manipulation	Portion size	Energy density^1^ (kJ/100g)
Soft hamburger	Bread, hamburger meat, tomato slices, ketchup	Soft bread	4 units ( = 700 g)	856
Hard hamburger	Bread, hamburger meat, tomato slices, ketchup	Hard bread	4 units ( = 700 g)	849
Soft rice salad	Rice, Chinese cabbage, carrot, white cabbage, paprika, parsley	Risotto rice Boiled vegetables	600 g	349
Hard rice salad	Rice, Chinese cabbage, carrot, white cabbage, paprika, parsley	White rice Raw vegetables	600 g	351

1 The energy densities were calculated from the used ingredients according to the Dutch Food Composition Database (NEVO, version 2011/3.0).

Dinner was served as a homogenous meal that consisted of 60% noodles, 10% chicken, 30% vegetables (onion, cucumber, radish, Chinese cabbage, bean sprout, garlic, chili pepper). The energy density calculated from the used ingredients was 463 kJ/100g, according to the Dutch Food Composition Database (NEVO, version 2011/3.0). Women were served 800 g and men 1000 g. The ad libitum intake was calculated by subtracting the weight of the left-overs from the weight of the initial served foods.

### Procedure for subjects

Subjects came on two separate days to the laboratory to consume lunch and dinner on the same day. When subjects came to the lunch sessions, they were given oral instructions about the procedure of the lunch. Thereafter, they were seated in sensory booths and received further instructions and questions via a computer screen.

Subjects were instructed to eat in their normal way and to eat as much until they felt “comfortably full” from the hamburgers and rice salad. They were instructed to take bites of the “whole” hamburger, and not to eat the meat or bread alone. They were served 150 ml of water. Exactly five hours after lunch, subjects returned to the laboratory to consume an ad libitum meal at dinnertime. They were again served 150 ml of water.

Before and after ad libitum intake of the lunch and dinner, subjects rated their feelings of hunger, fullness and thirst. In addition, subjects rated their perceived pleasantness and desire-to-eat for the presented foods after taking one bite of each. All questions were answered on a 100 mm visual analogue scale (VAS) that was scaled from “not at all” (0) to “very much” (100). Despite some limitations of the usage of VAS, we used this the subjective ratings in the present study, which is in line with previous reported research in this area [30], [31]. After the ad libitum intake at lunch, subjects were asked to rate the hamburgers and rice salad for a series of pre-defined sensory characteristics.

### Sensory characteristics

Subjective ratings of sensory characteristics were used to validate that subjects could perceive changes due to the hardness of the test foods. These included hardness, dryness, and chewiness intensity. All aspects were rated on a 100 mm VAS. The question that referred to hardness was: “How hard was the texture of the hamburger/salad?” from “very soft” at the left end (0) to “very hard” at the right end (100). The question that referred to dryness was: “How dry was the texture of the hamburger/salad?” from “very liquid” at the left end to “very dry” at the right end. The questions that referred to chewiness intensity was: “How strong was the chewiness intensity of the hamburger/salad?” from “very weak” to “very strong”.

### Oral processing characteristics

Oral processing characteristics were measured for 36 of the 50 subjects by using video records. Video recordings of 14 subjects were missing due to technical problems with the recordings on a test day. Video recordings were collected using a camera that was placed approximately 30 cm in front of the participants in the sensory booths. From these video recordings, the number of bites, the number of chews and the duration of the bites in the mouths (from the start of a bite until the last swallow before taking a new bite) were measured for consumption of hamburgers and rice salads separately. Two different experimenters coded independently all videos and the average was calculated when coding reliability was at least 80% for each variable. Whenever agreement was lower than 80%, which occurred in <5% of the coded videos, the two experimenters watched the video together and re-coded the data together. The average bite size (g/bite) per subject was calculated by dividing the ad libitum intake of a food-item (hamburger or salad) by the total number of bites of that food-item. The oral duration per bite per subject was calculated by measuring the time from the start of the bite until the bite was swallowed. The total oral duration (s) needed to consume a food-item was calculated as the sum of all oral durations of the bites of that food-item. The oral duration per gram was calculated by dividing the total oral duration per food-item by the ad libitum intake (g) of that food-item. Likewise, the chews per gram were calculated in the same manner. The overall eating rate (g/min) of the lunch was calculated by dividing the ad libitum intake (g) of both food items by the total duration (min) of the lunch.

### Standardization of satiety

To standardize the satiety state, subjects always started both lunch sessions at the same time. Dinner started exactly five hours after lunch. Subjects were instructed to consume the same breakfast at least three hours before lunch on each of the two test days. In addition, subjects were instructed to only drink water or coffee or tea without milk and sugar before lunch, and between lunch and dinner. After lunch, subjects answered questions about what they ate for breakfast and if they ate or drank between breakfast and lunch. After dinner, subjects answered questions about whether they ate or drank between lunch and dinner.

### Statistical analyses

Effect of hardness on intake, appetite ratings, hedonic ratings, sensory ratings, and oral processing characteristics were assessed in generalized linear models that included subject. Spearman's rank correlation coefficients were calculated for hedonic ratings, sensory ratings, and oral processing characteristics vs. food intake. Differences between correlation coefficients of softer and harder versions of the food-items were tested by Fisher's (*z*) tests. Statistical analyses were performed using SAS version 9.1.4 (SAS Institute Inc., Cary, NC, USA). Data were presented as means ± SDs. P-values of <0.05 were considered significant.

## Results

### Food and energy intake

**Figure 1:**
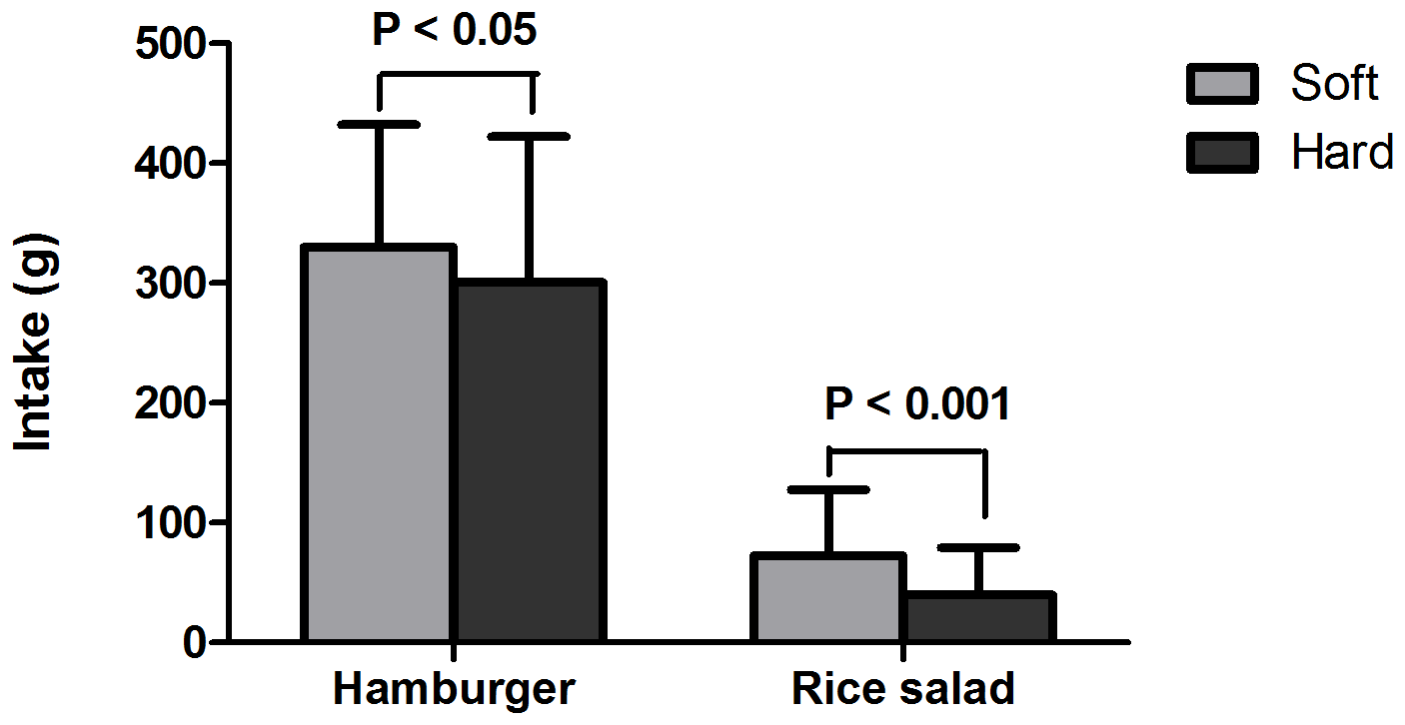
Food intake at lunch of soft and hard foods, n = 50 (means + SD). Total is the sum of hamburger and rice salad in either soft or hard versions.

**Figure 2:**
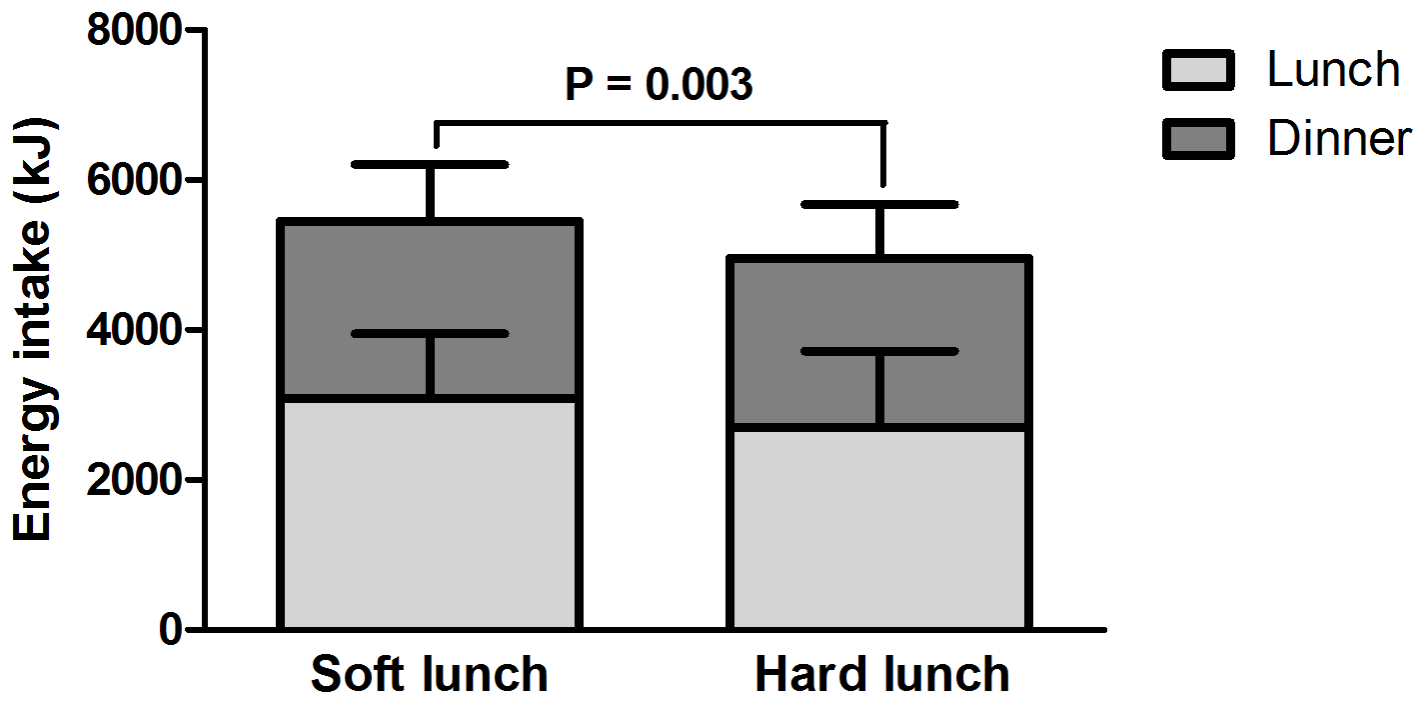
Energy intake at lunch and dinner, n = 50 (means and SD).

The hard hamburger led to 9% ( = 30 g) lower intake compared to the soft hamburger (P = 0.027) (Figure 1). The hard rice salad led to 17% ( = 33 g) lower intake compared to the soft rice salad (P<0.001). In total, the lunch with the harder foods led to a 16% ( = 63 g) lower intake compared to the lunch with softer foods (P<0.001). Energy intake of the hard hamburger was 274 kJ lower than the soft hamburger, and energy intake of the hard salad was 114 kJ lower than the soft salad. This was equal to a total reduction in energy intake of 13% (388 kJ) at lunch (P = 0.001) (Figure 2). The overall eating rate of the lunch with hard foods was ∼32% lower than that of the lunch with soft food, 25±7 g/min vs. 37±11 g/min, respectively (P<0.001). Water consumption was 110±60 g in the lunch with soft foods and 128±76 g in the lunch with hard foods (P = 0.09).

Energy intake at dinner was not different after both test lunches (Figure 2). Intake at dinner was 2367±751 kJ (511±162 g) after the lunch with soft foods, and was 2261±712 kJ (488±154 g) after the lunch with hard foods (P = 0.16). This means that the reduction in energy intake at lunch was not compensated for at dinner and led to a sustained reduction in energy intake of 9% over the two meals (494 kJ) (Figure 2). Water consumption at dinner was 126±76 g after lunch with soft foods and 114±45 g after the lunch with hard foods (P = 0.26).

### Appetite ratings

Ratings of hunger and fullness from both before and after lunch did not differ between the lunches with soft or hard foods (Table 2). Likewise, hunger and fullness before and after dinner did also not differ between both conditions. In addition, ratings of thirst before and after lunch and before and after dinner did not differ between both conditions (P>0.25) (data not shown).

**Table 2:** Hunger and fullness ratings before and after lunch and dinner.^1^

		Soft lunch	Hard lunch	P
Lunch	Hunger			
	Before	68±18	66±16	0.39
	After^2^	12±14	13±14	0.77
	Fullness			
	Before	22±18	23±20	0.73
	After^2^	80±16	78±12	0.51
Dinner	Hunger			
	Before	64±21	62±18	0.51
	After^2^	10±12	8.3±7.3	0.30
	Fullness			
	Before	32±20	32±20	0.92
	After^2^	82±16	83±11	0.41

1 Values are means ± SD, n = 50.

2 All ratings of hunger and fullness ratings after lunch and dinner were significantly different compared to the ratings before lunch and dinner, respectively (P<0.001).

### Hedonic and sensory ratings and oral processing characteristics

The hard versions of hamburger and rice salad were rated higher in hardness, dryness, and chewiness compared to their soft versions (Table 3). The hard hamburger was rated as slightly lower in pleasantness than the soft hamburger, though both foods were still rated as positively pleasant (i.e., >60 on the 100 mm VAS). There were no differences in hedonic ratings for the rice salads.

**Table 3:** Hedonic and sensory ratings of the foods.^1^

	Hamburger		P	Rice salad		P
	Soft	Hard		Soft	Hard	
Hedonic ratings						
Pleasantness	73±17	65±18	0.006	24±18	23±17	0.85
Desire-to-eat	70±15	65±19	0.09	23±14	21±16	0.63
Sensory ratings						
Hardness	26±17	73±15	<0.001	30±20	54±24	<0.001
Dryness	44±17	72±16	<0.001	36±20	67±20	<0.001
Chewiness	41±17	74±14	<0.001	35±18	51±22	<0.001

1 Values are means ± SDs, n = 50.

**Figure 3:**
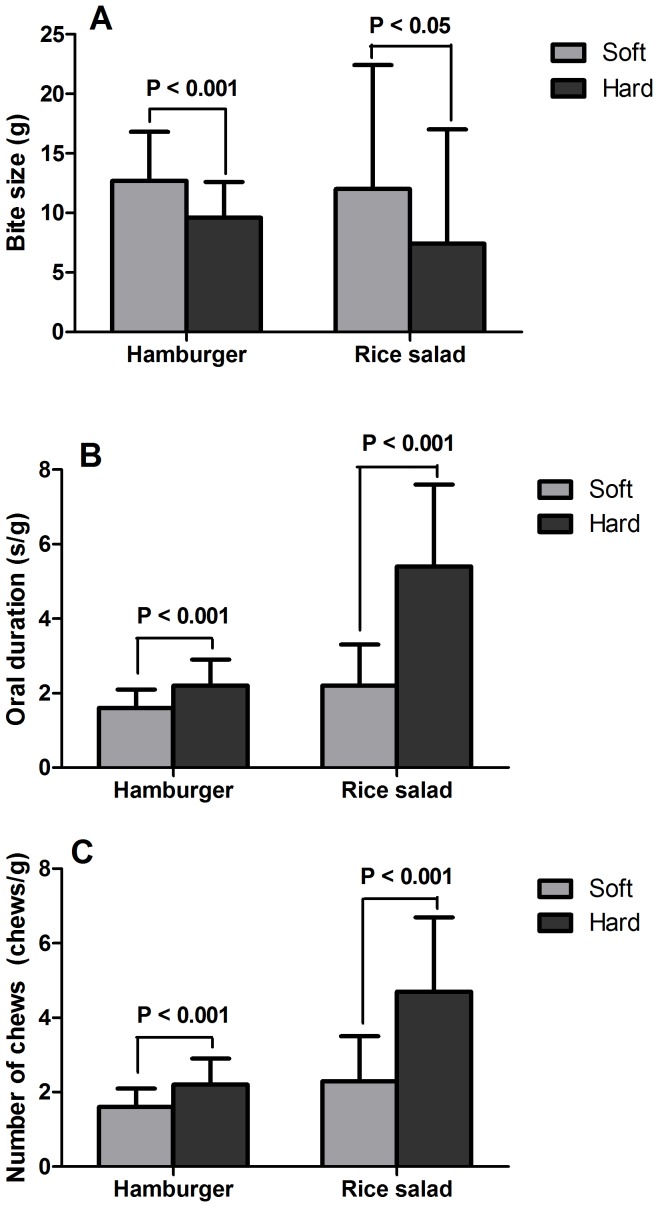
Differences in oral processing characteristics of soft and hard foods, n = 36 (means and SD); bite size (g) (A), oral residence duration (s/g) (B) and chews (no of chews/g) (C).

Results of the oral processing data show that the hard foods were consumed with smaller bites (Figure 3A), longer oral duration per gram food (Figure 3B) and more chews per gram food (Figure 3C).

### Correlations between food intake vs. hedonic ratings, sensory ratings and oral processing characteristics

Correlation coefficients between the hard and soft versions of the foods were not significantly different and therefore pooled together in all correlations described below. Food intake was positively correlated with the desire-to-eat, the pleasantness, and the bite size (Table 4). Oral duration per gram and chews per gram were negatively correlated with food intake. The sensory ratings for hardness, dryness, and chewiness intensity were negatively correlated with intake of rice salad, though these correlations were not found to be significantly correlated with hamburger intake. Overall, the sensory and oral processing characteristics were more strongly correlated with the intake of the rice salad than intake of the hamburger.

**Table 4:** Spearman's correlation coefficients between food intake vs. hedonic ratings, sensory ratings, and oral processing characteristics.

	Hamburger	Salad
	Intake (g)	Intake (g)
Hedonic ratings^1^		
Pleasantness	0.16	0.37***
Desire-to-eat	0.33***	0.42***
Sensory ratings^1^		
Hardness	−0.13	−0.32***
Dryness	−0.12	−0.41***
Chewiness intensity	−0.02	−0.26**
Oral processing characteristics^2^		
Bite size (g)	0.35**	0.52***
Oral duration per gram	−0.34**	−0.57***
Chews per gram	−0.22	−0.52***

1 n = 50.

2 n = 36.

** P<0.01, *** P<0.001.

### Correlations between sensory ratings and oral processing characteristics

As expected, the ratings for the sensory attribute hardness was positively correlated with the chews per gram (Rho  = 0.46 for hamburger, Rho  = 0.26 for salad, P<0.05), oral duration per gram (Rho  = 0.49 for hamburger, Rho  = 0.28 for salad P<0.05), and negatively with bite size (Rho  = −0.38 for hamburger, Rho  = −0.25 for salad P<0.05). Chewiness intensity for hamburger was positively correlated with oral duration per gram in hamburger (Rho  = 0.39, P<0.001), and negatively with bite size in hamburger (Rho  = −0.34, P = 0.003), these correlations were not found for salad (all P-values >0.22).

The chews per gram were strongly positively correlated with the oral duration per gram (Rho  = 0.93 for hamburger, Rho  = 0.97 for salad, P<0.001). Bite size was negatively correlated with chews per gram (Rho  = −0.40 for hamburger, Rho  = −0.74 for salad, P<0.001), and oral duration per gram (Rho  = −0.56 for hamburger, Rho  = −0.80 for salad, P<0.001).

## Discussion

The present study shows that harder foods led to lower overall food intake compared to softer foods (∼16% in gram and ∼13% in energy) served at lunchtime. Subjects felt equally full after both lunches despite a 13% difference in energy intake. The reduction in energy intake at lunch was not compensated for at dinner. This result indicates that differences in hardness in food led to a sustained reduction in energy intake over the course of the day.

The reduction in intake of the hard foods is likely to be explained by its effect on oral processing characteristics. The hard foods were perceived as harder, dryer and chewier than the soft foods (Table 3). Consumption of the hard foods resulted in slower eating rate with smaller bite sizes, longer oral residence durations, and more chewing per gram food. It has already been shown that these oral processing characteristics affect the amount of food intake in studies were these characteristics were manipulated explicitly by instructions on the frequency of chewing [27], [28], or experimentally by using fixed bite sizes and oral residence durations [23], [24], [26]. In the present study, subjects ate in response to the foods presented and did so naturally, without an explicit intention or conscious awareness that they were eating less.

Changing food texture is an efficient way to influence oral processing characteristics and food intake. However, very little research has been done in this field. A study by Zijlsta et al. [32] compared three types of solid foods (luncheon meat, meat replacer and candy) in soft and hard versions. Differences in hardness did not affect ad libitum intake in this study [32]. It may be that the textural differences in their study were too subtle to produce meaningful differences in oral processing that could affect ad libitum intake. The eating rate between the soft and hard versions was only different for the luncheon meat (∼16%), but not for the other two foods [32]. Forde et al. [33] have shown that “mashed” food increased the eating rate by approximately ∼20% compared to the “whole” version of the same food items. This led to a ∼10% difference in food intake in grams and was primarily driven by the vegetable component of the meal. The results of the present study showed that the eating rate was ∼32% lower for the lunch with hard foods. In addition, the oral duration per gram food (s/g) was about 40% higher in the harder version of hamburger and 105% higher in the harder version of rice salad compared to their softer versions. These differences were large enough to produce significant reductions in energy intake for the harder versions of both the hamburger and rice salad.

The mean difference in food intake between the soft and hard versions was larger for the rice salad than for the hamburger. This is possibly the result of the larger differences in oral processing characteristics between the soft and hard versions of salad, like the oral duration per gram and chews per gram. Oral processing characteristics were also more strongly correlated to food intake in the salad compared to the hamburger. It is possible that visual cues [34], and pre-planning [35] were more important in intake for the hamburger than the salad. It was easier for subjects to accurately monitor how much they consumed for the four presented hamburgers since they were pre-defined units, whereas it may have been more difficult for the 600 g of rice salad. Subjects may have had a tendency to consume whole units of the hamburger. This indicates that when texture modifications are made across different food items, the impact on oral processing behaviour and energy intake may differ depending on the nature of the food and the habitual manner in which it is consumed.

We chose to use similar ingredients for both the soft and hard version of the test foods (Table 1), in an effort to keep the palatability, macronutrient composition, and energy density equivalent. Despite these small differences in ingredients, the hard version of the hamburger was rated as slightly lower for pleasantness than the soft version of the hamburger. The results of the present study show that oral processing characteristics, but not pleasantness, were strongly correlated with hamburger intake. We therefore conclude that oral processing characteristics explain the reduction in intake of the hard hamburger, rather than the small difference in pleasantness.

With regard to the high prevalence of obesity, more attention is needed for the satiating capacity of foods. We found that hard foods lead to lower food intake, due to consumption with smaller bites size, higher chewing activity and longer oral residence durations. To get more insight in different textures of foods and the effort and time required for oral processing, Hutchings and Lillford [36] previously proposed the food oral process model to explain the breakdown path for different food types. In this model, food oral breakdown is defined on three distinct dimensions of degree of structure, degree of lubrication needed for swallow, and the time required in mouth to process the bite into a bolus that can be swallowed. A better understanding of the physical-chemical processes involved in the trajectory of oral breakdown may help in the design of foods with longer oral residence durations and greater requirements for chewing that result in earlier satiation and reduced energy intake.

This study shows that hard foods led to reductions in energy intake that sustain over the subsequent meal. However, it is not clear whether consumption of hard foods will produce sustained reductions in energy intake over longer periods. One study showed a reduction in food intake over two days when subjects took smaller bite sizes, whereas the changes in hunger and fullness did not differ from normal intake [37]. Other studies suggest that humans do not compensate well for moderate changes in energy intake over multiple days [38]–[40]. These findings encourage the idea that achieving satiation earlier through subtle changes in food properties could lead to decreased energy intake over the longer term. The next challenge will be to investigate whether changes in food texture can be used to produce sustainable reductions in energy intake over consecutive days.
